# Supplementary material for: Ulinastatin in the treatment of radiotherapy-induced oral mucositis in locoregionally advanced nasopharyngeal carcinoma: a phase 3 randomized clinical trial
Source: Nat Commun. 2025 Mar 23;16:2848. doi: 10.1038/s41467-025-57884-6 (PMC11930952; doi:10.1038/s41467-025-57884-6)
Supplement: Supplementary file 1 — Supplementary Information [file 41467_2025_57884_MOESM1_ESM.pdf]

Supplementary information

Contents

Supplementary Methods
Description of the guidelines for intensity-modulated radiation therapy in this trial....3
Supplementary Tables
Supplementary Table 1. List of Particiating Centers....5
Supplementary Table 2. Compliance to concurrent chemotherapy and radiotherapy....6
Supplementary Table 3. The onset times and duration of grade 3 RTOM....7
Supplementary Table 4. The rate of recovery from grade 3 RTOM during CCRT....8
Supplementary Table 5. Response to The Treatment....9
Supplementary Table 6. Patterns of Failure....10
Supplementary Table 7. Salvage Treatments After Relapse....11
Supplementary Figures
Supplementary Figure 1. Comparison of onset times and duration of grade 3 RTOM between two groups....12
Supplementary Figure 2. Survival Curves in UTI group and control group..13
Supplementary Note . Study Protocol....14
Statistical Analysis Plan....27
CONSORT checklist....29

Supplementary Methods

Description of the guidelines for intensity-modulated radiotherapy

All eligible patients in the present trial received intensity-modulated radiotherapy. In general, all patients were immobilised in the supine position with a thermoplastic mask used to cover the head, neck, and shoulder. Both non-enhanced computed tomography (CT) (for dose calculation) and contrast-enhanced CT (for target delineation) images) were obtained from the vertex to 2 cm below the sternoclavicular joint, with 3-mm slices.

The target volumes were defined in accordance with the International Commission on Radiation Units and Measurements Reports 50 and 62. We defined the gross tumour volume (GTV; including GTVnx and GTVnd) as the gross tumour determined by the physical examination, endoscopic findings, and imaging (including magnetic resonance imaging [MRI] and positron emission tomography [PET]-CT, if available) before concurrent chemoradiotherapy. GTVnx represented the sum of the enlarged retropharyngeal nodes and the primary tumour volume, while GTVnd represented the volume of the involved gross cervical lymph nodes. The GTVnx plus a 5–10-mm margin (2–3 mm posteriorly if adjacent to the spinal cord or brain stem) defined the high-risk clinical target volume (CTV1), which included the whole nasopharynx and the high-risk sites of microscopic extension. The CTV1 plus a 5–10-mm margin (2–3 mm posteriorly if adjacent to the spinal cord or brain stem) defined the low-risk clinical target volume (CTV2), which encompassed the low-risk sites of microscopic extension, such as the retropharyngeal nodal regions, parapharyngeal space, clivus, foramen lacerum, sphenoid sinus, pterygopalatine fossae, oval foramen, pterygoid fossae, posterior parts of the nasal cavity, the cervical level containing the involved lymph nodes, the elective neck area from level II to Vb, and the supraclavicular fossae. Level Ib was subjected to electively irradiation if: (1) there was involvement of level Ib lymph nodes; (2) level IIa lymph nodes with extracapsular extension or a diameter ≥ 2 cm was noted or there was bilateral involvement; (3) the ipsilateral neck had extensive nodal disease; and (4) the soft or hard palate, oral cavity, or ipsilateral nasal cavity were grossly involved. A threedimensional margin of 3–5 mm was added to the delineated target volume to compensate for internal organ motion and treatment set-up uncertainties, thus forming the planning target volume (PTV).

The prescribed dose was 68-72 Gy to PTVnx (Planning target volume of the primary tumor), 64-68 Gy to GTVnd, 60- 64Gy to PTVnd and PTV1 (Planning target volume 1), and 54-58 Gy to PTV2 (Planning target volume 2)

in 30-32 fractions. The radiation dose could be adjusted moderately according to the tumour volume. The normal tissue dose constraints are listed in the following table. All plans were generated by a team of dosimetrists using a wholefield (including neck radiation) simultaneous integrated boost technique. In general, when critical normal tissues (e.g., brain stem and spinal cord) were adjacent to the highdose target volumes, the target volume coverage could be compromised to keep these critical normal tissues within the dose constraints. When other normal tissues of lower priority were adjacent to the high-dose target volumes, the dose to these tissues was kept as low as possible without compromising the target coverage. The trade-off between covering the target volume and protecting the normal tissues in each case was discussed and decided upon by the research team.

Supplementary Tables

Supplementary Table 1. List of Particiating Centers

Center	Principal investigator	UTI group (n=89)	Control group (n=90)
Sun Yat-Sen University Cancer Center	Chong Zhao	72 (49.0%)	75 (51.0%)
Zhongshan City People's Hospital	Feng Lei, Yijing Ye	11 (61.1%)	7 (38.9%)
The First People's Hospital of Foshan	Xuefeng Hu	0 (0)	2 (100.0%)
The Third Affiliated Hospital of Sun Yat-sen University	Guanzhu Shen	0 (0)	5 (100.0%)
The Fifth Affiliated Hospital of Sun Yat-sen University	Zhigang Liu	6 (85.7%)	1 (14.3%)

Data are n (%).

Supplementary Table 2. Compliance to concurrent chemotherapy and radiotherapy

	UTI group (n=89)	Control group (n=90)
Patients receiving concurrent chemotherapy	89	90
Number of cycles of concurrent chemotherapy		
3 cycles	1	1
2 cycles	88[a]	89
Patients receiving RT	89	90

[a] One patient received 1 cycle cisplatin and 1 cycle carboplatin (due to grade 2 nephrototoxicity) .
Abbreviations: UTI=Ulinastatin.

Supplementary Table 3. The onset times and duration of grade 3 RTOM[a]

	UTI group	Control group
The onset times of grade 3 RTOM	26.00 [19.00, 33.00]	32.00 [20.50, 36.00]
The duration of grade 3 RTOM	12.00 [7.00, 18.00]	15.00 [7.50, 25.50]

Data are Median (IQR), days
[a] The onset times and duration of grade 3 RTOM were recorded from the start of radiotherapy for each patient until the end of the seventh week.
Abbreviations: UTI=Ulinastatin; RTOM=radiotherapy-induced oral mucositis; IQR= interquartile range.

Supplementary Table 4. The rate of recovery from grade 3 RTOM during CCRT

	**UTI group** no./total no. (%)	**Control group** no./total no. (%)
The rate of recovery from grade 3 RTOM	9/23 (39.1)	4/37 (10.8)

Abbreviations: UTI=Ulinastatin; RTOM=radiotherapy-induced oral mucositis; CCRT=concurrent chemoradiotherapy.

Supplementary Table 5. Response to The Treatment[a]

	UTI group (n=89)	Control group (n=90)
Complete response	43 (48.3%)	37 (41.1%)
Partial response	45 (50.6%)	53 (58.9%)
Stable disease	1 (1.1%)	0
Objective response rate	88 (98.9%)	90 (100.0%)

Data are n (%).
[a] Response to treatment was assessed according to RECIST 1.1 3 months after the end of radiotherapy.
Abbreviations: UTI=Ulinastatin.

Supplementary Table 6. Patterns of Failure

	UTI group (n=89)	Control group (n=90)
Local/regional relapse and distant metastasis	0	1 (1.1%)
Local and/or regional relapse only	7 (7.9%)	7 (7.8%)
Local relapse	6 (6.7%)	5 (5.6%)
Regional relapse	1 (1.1%)	1 (1.1%)
Locoregional relapse	0	1 (1.1%)
Distant metastasis only	1 (1.1%)	5 (5.6%)
Distant metastatic sites		
Lung	1 (1.1%)	2 (2.2%)
Liver	1 (1.1%)	1 (1.1%)
Bones	0	4 (4.4%)
Non-regional lymph nodes[a]	0	1 (1.1%)
Others	0	0
Unknown	0	0
Deaths	3 (3.4%)	6 (6.7%)
NPC-related	1 (1.1%)	4 (4.4%)
Non-NPC-related	0	0
Unknown	2 (2.2%)	2 (2.2%)

Data are n (%).

[a] Includes mediastinal, celiac or axillary lymph nodes metastasis.

Abbreviations: UTI=Ulinastatin; NPC, nasopharyngeal carcinoma.

Supplementary Table 7. Salvage Treatments After Relapse

	UTI group (n=89)	Control group (n=90)
Treatments after locoregional relapse	7	8
Surgery +/- adjuvant chemotherapy or radiotherapy	2	1
Chemotherapy	-	-
Chemoradiotherapy	-	1
Anti-PD1 + chemotherapy or radiotherapy	3	6
Traditional Chinese medicine	-	-
Unknown	2	-
Treatments after distant metastasis	2	10
Surgery+/- adjuvant chemotherapy or radiotherapy	-	1
Chemoradiotherapy	1	2
Chemotherapy	-	2
Anti-PD1 +/- chemotherapy	1	5
Traditional Chinese medicine	-	-
Unknown	-	-

Abbreviations: UTI=Ulinastatin; anti-PD1, anti-programmed death 1 antibody.

Supplementary Figures

Supplementary Figure 1. Comparison of onset times and duration of grade 3 RTOM between two groups[a]

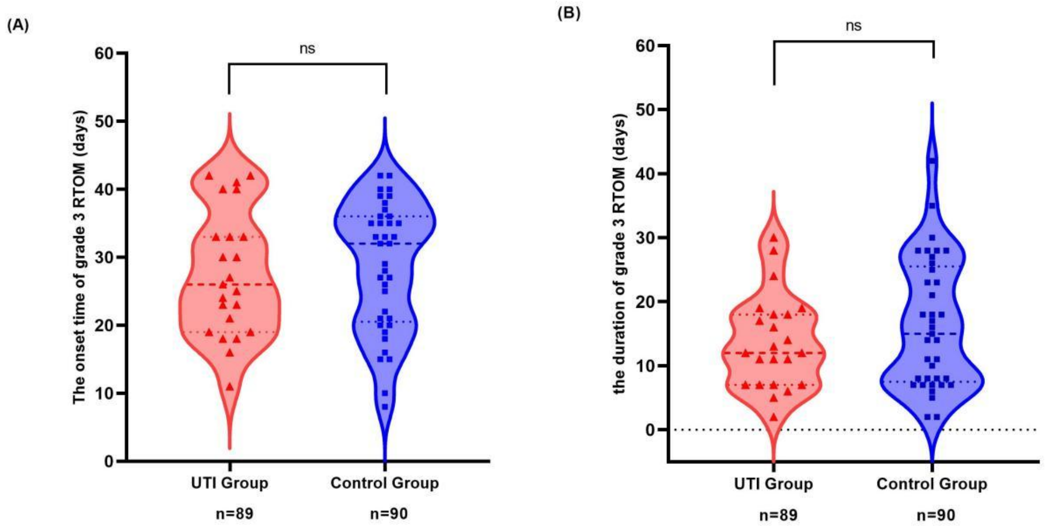

(A) The onset times of grade 3 RTOM in UTI group (23 patients) and control group (37 patients). There was no statistically significant difference in the onset times of grade 3 RTOM between two groups (ns, P = 0.621). P values were calculated by two-tailed, Mann – Whitney U test. (B) The duration of grade 3 RTOM in UTI group (23 patients) and control group (37 patients). There was no statistically significant difference in the onset times of grade 3 RTOM between two groups (ns, P = 0.393). P values were calculated by two-tailed, Mann – Whitney U test. All data in violin plots are represented as median value and quartile; each dot represented one sample. Source data are provided as a Source Data file.

[a] The onset times and duration of grade 3 RTOM were recorded from the start of radiotherapy for each patient until the end of the seventh week.

Abbreviations: UTI=Ulinastatin; RTOM=radiotherapy-induced oral mucositis.

Supplementary Figure 2. Survival Curves in UTI group and control group

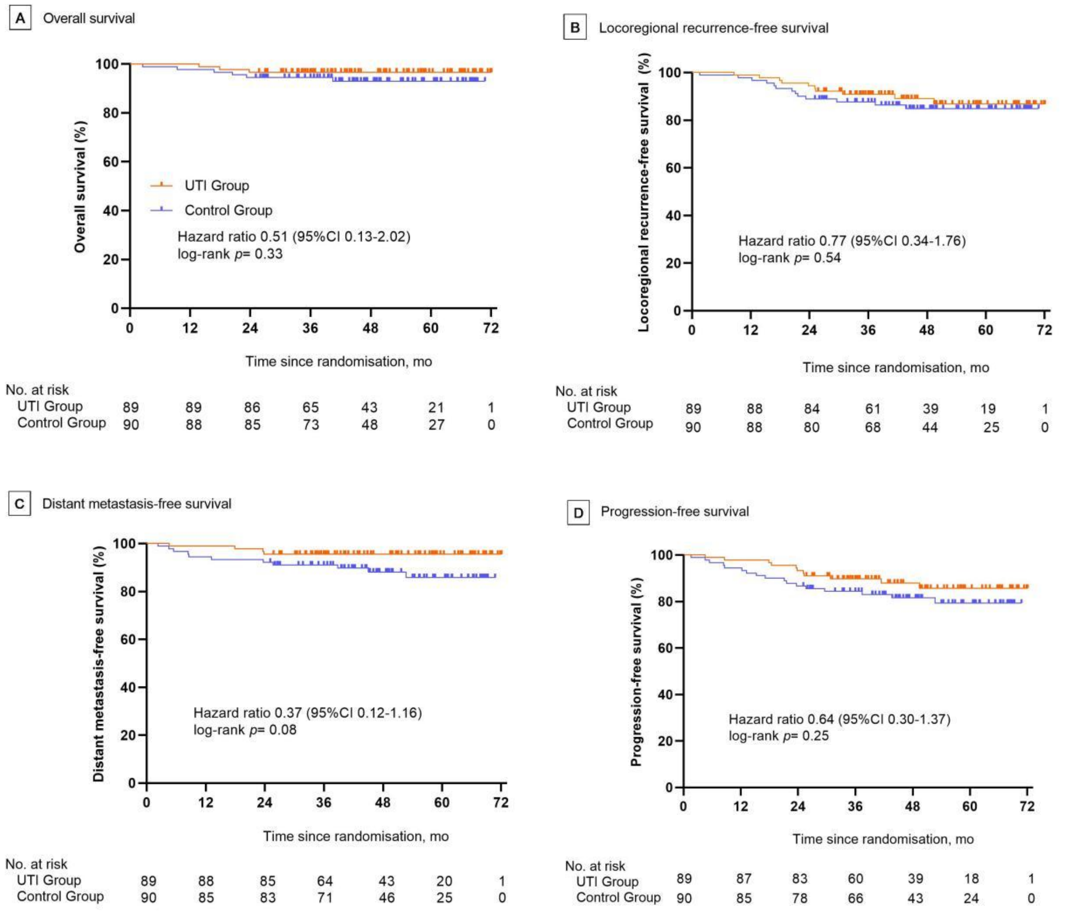

(A) Overall survival. (B) Locoregional relapse-free survival. (C) Distant metastasis-free survival. (D) Progression-free survival.

The statistical significance was assessed using the two-sided log-rank test, with the exact P-value displayed. Source data are provided as a Source Data file.

Abbreviations: UTI=Ulinastatin.

Supplementary Note. Study Protocol

The efficacy and safety of Ulinastatin in the prevention and treatment of radiotherapy-induced oral mucositis in locoregionally advanced nasopharyngeal carcinoma (LA-NPC): A multicenter, open-label, randomized controlled clinical trial

Principal investigator: Prof. Chong Zhao
Department of Nasopharyngeal carcinoma, Sun Yat-sen University Cancer Centre

Version: 1.1
Date: October, 2017

Country	Institution	Investigator
Foshan	The First People's Hospital of Foshan	Xuefeng Hu
Guangzhou	The Third Affiliated Hospital of Sun Yat-sen University	Guanzhu Shen
Zhuhai	The Fifth Affiliated Hospital of Sun Yat-sen University	Zhigang Liu
Zhongshan	Zhongshan City People's Hospital	Feng Lei, Yijing Ye

Contact: Lin Wang, Email: wangl1@sysucc.org.cn

TABLE OF CONTENTS

	Title page	14
	Table of Contents	15
1	Background	16
2	Study Objectives and Endpoints	17
2.1	Primary objective	17
2.2	Secondary objectives	17
2.3	Definition of endpoints	17
3	Patient Eligibility Criteria	18
3.1	Inclusion criteria	18
3.2	Exclusion criteria	18
4	Study Schema	18
5	Treatment Protocol	19
5.1	Radiotherapy	19
5.2	Concurrent chemotherapy	20
5.3	Ulinastatin therapy	21
5.4	General clinical management of RTOM	21
6	Baseline Assessment and Follow-up	22
6.1	Baseline assessment	22
6.2	Assessment during treatment	22
6.3	Follow-up schedule	22
6.4	Salvage treatment	23
7	Ethical and regulatory requirements	23
7.1	Ethical conduct of the study	23
7.2	Patient data protection	23
7.3	Ethics and regulatory review	23
7.4	Informed consent	23
7.5	Changes to the protocol and informed consent form	24
7.6	Audits and inspections	24
8	Statistical Analysis	24
8.1	Sample size calculation	24
8.2	Statistical analysis	25
9	References	25

LIST OF TABLES

Table 1 Delineation of planning target volumes (PTVs) 19
Table 2 Delineation and dose limits of organs at risk (OARs) 20

LIST OF FIGURES

Figure 1 Study flow chart 18

1 Background

radiotherapy-induced oral mucositis (RTOM) refers to the non-specific inflammatory reaction of oral mucosa after exposure to a certain dose of radiation. The main mechanisms are as follows : (1) Radiation directly acts on the DNA of oral/oropharyngeal mucosal epithelial cells, induces local apoptosis, overactivation of inflammatory pathways, and a large number of inflammatory factors (TNF-a, TGF-β, IL-1β, IL-6, MPO, COX2, etc.) are released; (2) Changes in enzyme activity of oral/oropharyngeal mucosal epithelial cells, apoptosis of vascular endothelial cells and fibroblasts.[1] In addition, the immune system is suppressed in patients with radiotherapy and chemotherapy, causing RTOM to develop into specific infectious inflammation, further aggravating the degree of local inflammation. RTOM is a common side effect of radiotherapy in head and neck tumors, especially nasopharyngeal carcinoma, with an incidence of 50%-100%,[2, 3] which is related to radiation dose and segmentation method. It is also related to the type and dose of chemotherapy drugs used in combination with chemotherapy. In recent years, radiotherapy has entered the era of intensity-modulated radiation therapy (IMRT), which is beneficial to local area control and protection of surrounding important tissues and organs. However, this technique increases the total and fractional doses of oropharyngeal irradiation, resulting in a significant increase in the incidence and severity of RTOM. On the one hand, moderate to severe RTOM can cause oral pain in patients, affect eating, increase the risk of malnutrition and the incidence of treatment interruption, prolong hospital stay, and reduce the efficacy of radiotherapy and chemotherapy. On the other hand, it is easy to develop from non-specific inflammation to infectious inflammation, which increases the difficulty of treatment and increases the use of antibiotics. Therefore, the study of effective prevention and treatment of RTOM is of great significance for improving treatment compliance, reducing the use of antibiotics, and ultimately improving the therapeutic effect and quality of life of patients.

In the past decade, domestic and foreign scholars have carried out a large number of studies on the prevention and treatment of RTOM, such as applying non-steroidal anti-inflammatory drugs, mucosal protective agents, granulocytic-macrophage colony stimulating factors, etc.[4-7] However, there is still a lack of effective prevention and treatment of RTOM, and symptomatic treatment such as local analgesia is still the main treatment. Therefore, it is necessary to further search for effective drugs.

Ulinastatin is a kind of glycoprotein that can inhibit the activity of a variety of proteolytic enzymes. At the same time, it also has the site of membrane receptor recognition and binding, which has the role of stabilizing membrane and lysosomal membrane. Therefore, Ulinastatin can reduce the release of inflammatory mediators and block inflammatory responses. Several cell experiments, animal experiments and clinical studies have shown that Ulinastatin can play an important role in suppressing inflammatory mediators

by modulating inflammation related factors and regulating inflammatory response modulation signaling-related factors.[8-11] Therefore, we hypothesized that Ulinastatin may play the same role in RTOM. To further evaluate the efficacy of Ulinastatin in the treatment of oral mucositis, we conducted a multicenter prospective randomized trial of Ulinastatin for the prevention and treatment of acute oral mucositis in patients undergoing concurrent chemoradiotherapy (CCRT) for NPC.

2 Study Endpoints

2.1 Primary Endpoint

To investigate if the addition of Ulinastatin (UTI group) reduces the incidence of grade ≥ 3 acute radiotherapy-induced oral mucositis (RTOM) among patients with locoregionally advanced nasopharyngeal carcinoma (LA-NPC) who receive concurrent chemoradiotherapy (CCRT).

2.2 Secondary endpoints

1. cumulative rate of grade ≥ 3 RTOM from 0 to 7 weeks of radiotherapy ;
2. onset time of grade ≥ 3 RTOM；
3. duration of grade ≥ 3 RTOM from 0 to 7 weeks of radiotherapy；
4. recovery rate from grade 3 RTOM;
5. severe oral pain;
6. safety;
7. completion rate of planned CCRT and the incidence of radiotherapy interruption (radiotherapy interruption ≥ 5 days);
8. overall survival (OS), locoregional relapse-free survival (LRRFS), distant metastasis-free survival (DMFS) and progression-free survival (PFS).

2.3 Definition of endpoints

1. recovery rate from grade 3 RTOM：proportion of patients with grade ≥ 3 RTOM who recovered to grade ≤ 2 during CCRT.
2. OS: durations were calculated from the date of randomization to the date of last follow-up or death from any cause.
3. LRRFS, durations were calculated from the date of randomisation and the date of locoregional recurrence, or death from any cause.
4. DMFS: durations were calculated from the date of randomisation and the date of distant metastasis, or death from any cause.
5. PFS: durations were calculated from the date of randomisation to the date of locoregional recurrence, distant metastasis, or death from any cause, whichever occurred first.

3 Patient Eligibility Criteria

3.1 Inclusion criteria

1. Patients with newly diagnosed, pathologically confirmed NPC;

2. Patients with TNM stage III or stage IVa according to the staging system of the 8th American Joint Committee on Cancer/Union for International Cancer Control (AJCC/UICC);
3. Patients between 18 and 65 years;
4. Patients with adequate organ function (white blood cell count of ≥4.0×109/L; absolute 142 neutrophil count of ≥1.5×109/L; hemoglobin of ≥100g/L; platelet count of ≥100×109/L; 143 total bilirubin, aspartate aminotransferase and alanine aminotransferase of ≤1.5X the 144 upper limit of normal [ULN]; and creatinine clearance rate of ≥60 mL/min);
5. Patients with Karnofsky performance Status (KPS score) ≥ 80 points.

3.2 Exclusion criteria

1. Patients with distant metastases;
2. Patients with prior anti-tumor therapy;
3. Patients with metachronous or synchronous malignancy
4. Patients with drug or alcohol addiction;
5. Patients lacking capacity for providing informed consent;
6. Patients suffering from active systemic infections;
7. Patients concurrent pregnancy or lactation;
8. Patients suffering from severe comorbidities such as poorly controlled diabetes, hypertension, hepatitis and tuberculosis.

4 Study Schema

This is a prospective, multicenter, randomized, controlled trial. At least 176 patients with LA-NPC will be recruited and randomly assigned in a 1:1 ratio to UTI group or control group. The study flow chart is presented in Figure 1.

Figure 1 Study flow chart

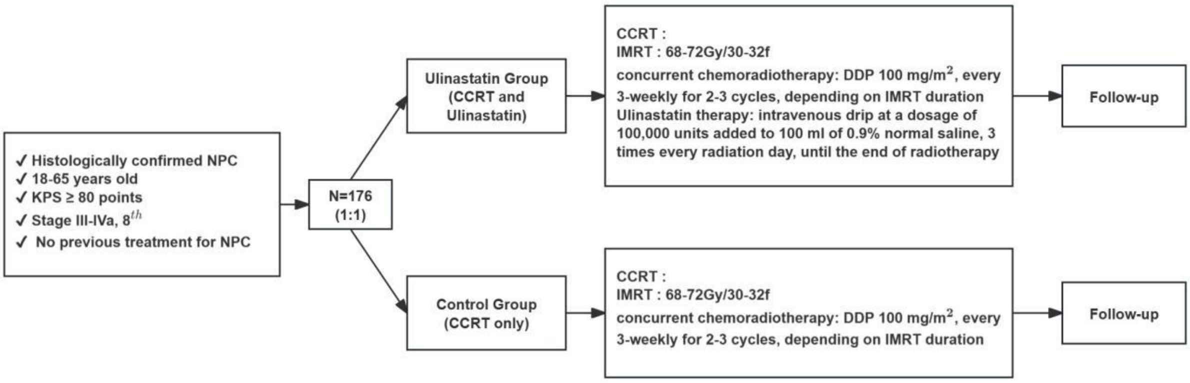

5 Treatment Protocol

5.1 Radiotherapy

5.1.1 Radiotherapy preparation

All patients are immobilized in the supine position using a thermoplastic mask that covered the head, neck, and shoulder. Both non-enhanced computed tomography (CT) (for dose calculation) and contrast enhanced CT (for target delineation) images will be obtained from the vertex to 2.0 cm below the sternoclavicular joint, with 3-mm slices.

5.1.2 Target volume delineation

The target volume delineation includes the GTVp, gross tumor volume of involved lymph nodes (GTVn), high-risk clinical target volume (CTV1) and low-risk clinical target volume (CTV2).

1. The GTVp and GTVn are defined according to the magnetic resonance imaging (MRI) images, as well as clinical and endoscopic findings.
2. The CTV1 is defined as the GTVp plus a 5-10 mm margin (2-3 mm margin posteriorly) to encompass the high-risk sites of microscopic extension and the whole nasopharynx.
3. The CTV2 is defined as the CTV1 plus a 5-10 mm margin (2-3 mm margin posteriorly) to encompass the low-risk sites, and the elective neck area (bilateral levels IIa-b, III, and Va are routinely covered for all N0 patients, whereas ipsilateral levels IV, Vb, or supraclavicular fossae are also included for N1-3 patients).
4. Level Ib will be electively irradiated if: level Ib LNs are involved, level IIa LNs have a diameter ≥3cm or extracapsular extension is present, extensive nodal disease is present on the ipsilateral neck, and the soft or hard palate, oral cavity, or ipsilateral nasal cavity are grossly involved.
5. Planning target volumes (PTVs) are generated automatically after delineation of tumor targets by a uniform expansion ranging from 3 mm (1 mm posteriorly), depending on immobilization and localization uncertainties, as shown in Table 1.

Table 1 Delineation of planning Target Volumes (PTVs)

PTVs	Margins
PTVp	GTVp+3mm (1mm posteriorly)
PTVn	GTVn+3mm
PTV1	CTV1+3mm (1mm posteriorly)
PTV2	CTV2+3mm (1mm posteriorly)
Brainstem_PRV	Brainstem+1mm
Spinal cord_PRV	Spinal cord+5mm
Optic nerve_PRV	Optic nerve+1mm

Optic chiasm_PRV	Optic chiasm+1mm

5.1.3 Prescribed dose to tumor target volumes

The prescribed doses are 68-72 Gy/30-32 fractions to PTVp, 60-68 Gy/30-32 fractions to PTVn, 60-64 Gy/30-32 fractions to PTV1, 54-58 Gy/30-32 fractions to PTV2.

The plan will be accepted if:

1. the prescribed dose encompasses at least 95% of the target volume;
2. no greater than 1% of the GTVp receives >95% of the prescribed dose;
3. the maximum dose point is located in the GTVp.

5.1.4 Delineation and dose constraints of organs at risk

All adjacent critical structures, including the brainstem, spinal cord, temporal lobes, lens, optic nerves and chiasm, parotid glands, temporomandibular joints, and mandibles will be carefully outlined. The maximum dose to these structures is kept within their dose limits, which are listed in Table 2.

Table 2 Dose limits of organs at risk (OARs)

**OARs**	**Dose limits**
Brainstem_PRV	Dmax ≤56Gy
Spinal cord_PRV	Dmax ≤45Gy
Temporal lobes_PRV	Dmax ≤60Gy
Pituitary	Dmax ≤50Gy
Lens	Dmax ≤5Gy
Optic nerve	Dmax ≤50Gy
Optic chiasm	Dmax ≤50Gy
Temporomandibular joints	Dmax ≤56Gy; D33 ≤45Gy
Mandible	Dmax ≤65Gy; D33 ≤45Gy
Parotids	Dmax ≤60Gy; D33 ≤35Gy

5.1.5 Radiotherapy delay

Radiotherapy will be delayed if patients had ≥grade 3 mucositis or skin reaction and resumed 215
only when these toxicities have recovered ≤grade 2.

5.2 Concurrent chemotherapy

5.2.1 Concurrent chemotherapy regimen

Cisplatin is given at a dose of 100mg/m^2 intravenouslyevery 3 weeks for 2 to 3 cycles depending on the duration of radiotherapy.

5.2.2 Dose modification

Dose modifications for hematological and non-hematological toxicities during CCRT will be assessed based on the nadir blood counts and acute toxicities of the preceding cycle.

Cisplatin dose will be reduced by one level (20% [20mg/m²]) if the patient suffers from the following toxicities:

1. grade 4 neutropenia, febrile neutropenia, neutropenic infection, or related toxicities lasting for more than 7 days;
2. grade 4 thrombocytopenia; 230
3. creatinine clearance of 40–60mL/min;
4. grade 2 neurotoxicity;
5. grade 3 gastrointestinal toxicity.

5.2.3 Chemotherapy discontinuation

Chemotherapy will be discontinued if the patient suffers from the following toxicities:

1. Grade 3 hepatotoxicity (aspartate aminotransferase, alanine minotransferase, or alkaline phosphatase ≥5.0 times ULN);
2. creatinine clearance of <40mL/min;
3. two episodes of grade 4 diarrhea;
4. grade 3 or higher neurotoxicity.

5.3 Ulinastatin therapy

Ulinastatin is given through intravenous drip at a dosage of 100,000 units added to 100 ml of 0.9% normal saline, 3 times every radiation day, until the end of radiotherapy.

The criteria for discontinuing Ulinastatin were as follows : 1. The patient refuse Ulinastatin treatment; 2. Allergic reaction during treatment; 3. Intolerance of associated toxicity.

5.4 General clinical management of RTOM

1. Strengthen oral care and education;
2. Oral treatment before radiotherapy: replace metal fillings with non-metal fillings, remove metal dentures and affected teeth according to the advice of dentists, etc.;
3. In the presence of mild pain (NRS pain score 1-3) and erythema of oral mucosa, non-opioid painkillers should be used to relieve pain and local sprays should be used to promote mucosal repair.
4. In the presence of moderate pain (NRS pain score 4-6) that does not interfere with transoral feeding, spotty oral ulcers, or scattered mucosal leukoplakia, dietary modifications were made to avoid stimulating or hard foods, and local analgesia with 0.5%-1.0% procaine gargle, systemic

analgesia with weak opioids, and aerosolised throat sprays (pramipexole, chymotrypsin, etc.) were administered.
5. In the presence of severe pain (NRS pain score 7-9) that interferes with oral intake, fused oral ulcers or fused mucosal leukoplakia, and bleeding due to minor trauma, liquid diet and intravenous nutrition should be supplemented as appropriate, local analgesia with 0.5%-1.0% procainine, systemic analgesia with strong opioids, and spray throat should be given. If there is a specific infection, anti-infection treatment should be given.
6. In the event of obvious spontaneous bleeding due to necrosis of oral mucosal tissue or life-threatening complications, radiotherapy and chemotherapy should be stopped, and total intravenous nutrition should be given, as well as the above-mentioned local pain relief, systemic pain relief, anti-infection and other comprehensive symptomatic supportive treatment.

6 Baseline Assessment and Follow-up

6.1 Baseline Assessment

Patients must undergo the following examinations to determine the stage and other baseline parameters within two weeks before randomization:
1. Medical history review, physical examination of the head and neck region, neurological assessment, KPS evaluation.
2. Nasal endoscopy and biopsy for histological confirmation of NPC.
3. Blood routine and biochemistry tests including full blood count, renal panel, liver panel, electrolyte panel, lipid panel, blood glucose and inflammatory factors panel.
4. MRI or CT imaging of the head and neck region.
5. 18F-FDG-PET-CT for distant staging.
6. If 18F-FDG-PET-CT is not performed, conventional workup such as CT of the thorax and abdomen and emission computed tomography.
7. Plasma EBV DNA assays, which will be centrally performed at the SYSUCC for standardization.

6.2 Assessment During Treatment

Routine physical examination, hematological and biochemical blood tests will be performed weekly during CCRT. Plasma EBV DNA assay will be performed on the last week of CCRT.The grade of RTOM and oral pain were recorded every week from the start of radiotherapy for each patient until the end of the seventh week.

Treatment compliance and treatment-related adverse events (TRAEs) will be assessed weekly during radiotherapy and at 3 and 6 months post-CCRT, or at the time of treatment discontinuation. TRAEs will be graded using the Common Toxicity Criteria for Adverse Events version 5.0 (CTCAE v5.0) for chemotherapy-related toxicities, and the Radiation Therapy Oncology Group (RTOG) radiation morbidity scoring criteria for radiotherapy-related toxicities.

6.3 Follow-up Schedule

All patients will be assessed by physical examination of the head and neck region and nasopharyngoscopy at the following intervals:

1. During years 1-3 post treatment: every three months.
2. During years 4-5 post treatment: every six months.

The nasopharyngoscopy will be performed 1 month post-CCRT to evaluate tumor response.

Hematological and biochemical blood tests, contrast-enhanced CT and/or MRI of the head and neck region performed, and CT of the thorax and abdomen will be performed 3 months post-CCRT, 6 months post-CCRT, and annually thereafter. ^{18}F-FDG-PET-CT can be used to detect distant metastasis at the clinician's discretion. Delayed TRAEs post-treatment will be assessed using the RTOG radiation morbidity scoring criteria six-monthly during the first year and yearly thereafter.

6.4 Salvage treatment

Biopsy or fine needle aspiration (FNA) should be used to confirmed disease recurrence, if the site is amenable for a biopsy. Salvage treatment for residual disease and/or tumor relapse, if detected, will be tailored on a case-by-case basis and NCCN Guidelines on Head and Neck Cancers and at the discretion of the physician-in-charge.

7 Ethical and Regulatory Requirements

7.1 Ethical conduct of the study

The study will be performed in accordance with ethical principles that have their origin in the Declaration of Helsinki and be consistent with International Conference on Harmonization (ICH)/Good Clinical Practice (GCP), applicable regulatory requirements and the Sun Yat-sen University Cancer Center (SYSUCC) policy on Bioethics and Human Biological Samples.

7.2 Patient data protection

The Informed Consent Form will be a separate document that complies with relevant data protection and privacy legislation. We will not provide any data concerning patients to any insurance company, any employer, their family members, general physician or any other third party, unless required to do so by law.

7.3 Ethics and regulatory review

An Ethics Committee, Independent Ethics Committee or Institutional Review Board (IRB) from SYSUCC, should approve the final study protocol, including the final version of the Informed Consent Form and any other written information and/or materials to be provided to the patients. The investigator will ensure the distribution of these documents to the applicable Ethics Committee, and to the study site staff.

7.4 Informed consent

The Principal Investigator(s) at each institution will:

1. Ensure each patient is given full and adequate oral and written information about the nature, purpose, possible risk and benefit of the study;
2. Ensure each patient is notified that they are free to withdraw from the study at any time;3. Ensure that each patient is given the opportunity to ask questions and allowed time to consider the information provided;
4. Ensure each patient provides signed and dated informed consent before conducting any procedure specifically for the study;
5. Ensure the original, signed Informed Consent Form(s) is/are stored in the Investigator's Study File;
6. Ensure a copy of the signed Informed Consent Form is given to the patient;
7. Ensure that any incentives for patients who participate in the study as well as any provisions for patients harmed as a consequence of study participation are described in the informed consent form that is approved by an Ethics Committee.

7.5 Changes to the protocol and informed consent form

Study procedures will not be changed without the mutual agreement of all coordinating investigators. If there are any substantial changes to the study protocol, then these changes will be documented in a study protocol amendment and where required in a new version of the study protocol (Revised Clinical Study Protocol). The amendment is to be approved by the relevant Ethics Committee and if applicable, also the national regulatory authority approval, before implementation. Local requirements are to be followed for revised protocols. If a protocol amendment requires a change to an institution's Informed Consent Form, the SYSUCC's Ethics Committee is to approve the revised Informed Consent Form before the revised form is used. If local regulations require, any administrative change will be communicated to or approved by each Ethics Committee.

7.6 Audits and inspections

The Ethics Committee will perform audits or inspections including source data verification. The purpose of an audit or inspection is to systematically and independently examine all study-related activities and documents, to determine whether these activities are conducted, and data are recorded, analyzed, and accurately reported according to the protocol, GCP, guidelines of the ICH, and any applicable regulatory requirements.

8 Statistical Analysis

8.1 Sample size calculation

In this study, the "power analysis and sample size" software (version 2018) was used to calculate the total sample size. According to previous studies, the

incidence of grade ≥ 3 oral mucositis in NPC treated by IMRT with cisplatin was about 40%.5, 26 Assuming that the incidence of grade ≥ 3 oral mucositis in the Ulinastatin group decreased to 20%, the significance test level α= 0.05, Power= 0.8, at least 80 cases in each group were estimated. At least 176 patients (88 cases per group) were required to be enrolled in this study based on a 10% dropout and loss of follow-up rate.

8.2 Statistical analysis

Patient demographics and clinical characteristics that are categorical variables will be summarized as frequencies with percentages, and continuous variables will be summarized as medians with inter-quartile ranges (IQR). Survival curves will be derived using the Kaplan-Meier method and compared using the log-rank test. Unstratified Cox proportional hazards regression models will be used to estimate hazard ratios (HRs). Corresponding 95% confidence intervals (CIs) will be based on the Wald test. The proportional hazards assumption will be tested by including time-dependent covariates in the Cox models within the PROC PHREG module. Survival rates at 3- and 5-year will be reported with corresponding 95% CIs calculated using the log(-log) transformation of survival probabilities. Median follow-up time will be estimated using the reverse Kaplan-Meier method. All statistical analyses were performed using the SPSS software, version 25.0 (IBM Corp., Chicago, IL, USA) and were 2-sided at a significance level of P <0.05. Figures will be drawn using GraphPad Prism version 7.0.0 (GraphPad Software, San Diego, CA).

9 References

1. Sonis ST. Mucositis: the impact, biology and therapeuticopportunities of oral mucositis[J]. Oral Oncol.2009, 45(12):1015-1020.
2. Lei Chen, Jun Ma,et al. Concurrent chemoradiotherapy plus adjuvant chemotherapy versus concurrent chemoradiotherapy alone in patients with locoregionally advanced nasopharyngeal carcinoma:a phase 3 multicentre randomised controlled trial. Lancet Oncol .2012,13: 163 – 71.
3. Ying Sun, Jun Ma,et al. Induction chemotherapy plus concurrent chemoradiotherapy versus concurrent chemoradiotherapy alone in locoregionally advanced nasopharyngeal carcinoma: a phase 3, multicentre,randomised controlled trial, Lancet Oncol. 2016 Nov,17(11):1509-1520.
4. Shao-Xiong Wu, Tian-Tian Cui , Chong Zhao, et al. A prospective, randomized, multi-center trial to investigate Actovegin in prevention and treatment of acute oral mucositis caused by chemoradiotherapy for nasopharyngeal carcinoma. Radiother Oncol. 2010 Oct,97(1):113-8.
5. Rajesh V. Lallaa, Linda E. Choquettea, et al. Randomized Double-blind Placebo-controlled Trial of Celecoxib for Oral Mucositis in Patients Receiving Radiation Therapy for Head and Neck Cancer. Oral Oncol. 2014 November,50(11): 1098–1103.

6. Eng-yen huang, Stephen wan leung, et al. oral glutamine to alleviate radiation-induced oral mucositis: a pilot randomized trial. Int. J. Radiation Oncology Biol. Phys, 2000,46(3):535–539.
7. M. Hejna, W.J. Ko¨ stler, et al. Decrease of duration and symptoms in chemotherapy-induced oral mucositis by topical GM-CSF: results of a prospective randomised trial. European Journal of Cancer. (2001) 37:1994–2002.
8. Pengtao Bao a,1, Wei Gao, et al. Effect of pretreatment with high-dose ulinastatin in preventing radiation-induced pulmonary injury in rats. European Journal of Pharmacology, 603 (2009), 114–119.
9. Pan Y, Fang H, et al. Ulinastatin ameliorates tissue damage of severe acute pancreatitis through modulating regulatory T cells. J Inflamm (Lond). 2017 Mar 20,14:7.
10. Huang SW, Guan XD,et al. Clinical study and long-term evaluation of immunomodulation therapy on trauma, severe sepsis and multiple organ dysfunction syndrome patients. Zhongguo Wei Zhong Bing Ji Jiu Yi Xue. 2006 Nov,18(11):653-6.
11. Liu D, Yu Z,et al. Effect of ulinastatin combined with thymosin alpha1 on sepsis: A systematic review and meta-analysis of Chinese and Indian patients. J Crit Care.2016VN.

Statistical Analysis Plan

1 Overview

The study is a prospective, multicenter, open-label, randomized, controlled clinical trial. The aim of this study is to evaluate the efficacy and safety of Ulinastatin for the prevention and treatment of radiotherapy-induced oral mucositis (RTOM) in locoregionally-advanced nasopharyngeal carcinoma (LA-NPC) patients who receive concurrent chemoradiotherapy (CCRT).

2 Sample size calculation

Sample size of this study is calculated using the Power and Sample Size Calculation (version 2018) software. Assuming that the incidence of grade ≥ 3 RTOM in the Ulinastatin group decreased to 20%, the significance test level α= 0.05, Power= 0.8, at least 80 cases in each group were estimated. At least 176 patients (88 cases per group) were required to be enrolled in this study based on a 10% dropout and loss of follow-up rate.

3 Definitions of the outcomes

3.1 Primary outcome

The primary outcome is the incidence of graded ≥ 3 acute RTOM in both groups.

3.2 Secondary outcomes

Secondary outcomes will include the following:

1. cumulative incidence of grade ≥ 3 RTOM from 0 to 7 weeks of radiotherapy ;
2. onset time of grade ≥ 3 RTOM;
3. duration of grade ≥ 3 RTOM from 0 to 7 weeks of radiotherapy;
4. recovery rate from grade 3 RTOM: proportion of patients with grade ≥ 3 RTOM who recovered to grade ≤ 2 during CCRT;
5. severe oral pain;
6. Treatment-related adverse events (TRAEs): TRAEs will be graded using the Common Toxicity Criteria for Adverse Events version 4.0 (CTCAE v5.0) for chemotherapy-related toxicities, and the Radiation Therapy Oncology Group (RTOG) radiation morbidity scoring criteria for radiotherapy-related toxicities.
7. Completion rate of planned CCRT and the incidence of radiotherapy interruption (radiotherapy interruption ≥ 5 days);
8. Overall survival (OS), defined as time from date of randomization to the date of last follow-up or death from any cause;
9. Distant metastasis-free survival (DMFS), defined as the time from the date of randomisation and the date of distant metastasis, or death from any cause;
10. Locoregional relapse-free survival (LRRFS), defined as the time from the date of randomisation and the date of locoregional recurrence, or death from any cause;
11. progression-free survival (PFS), defined as the time from the date of from

the date of randomisation to the date of locoregional recurrence, distant metastasis, or death from any cause, whichever occurred first.

4 Statistical analysis

4.1 Analysis principles

1) For two-sided tests, the nominal level of type I error (α) will be 0.05 and the confidence level for two-sided confidence intervals (CI) will be 95%;
2) There will be no imputation of the missing values. The number of observations used in the analysis will be reported;
3) Intention-to-treat principle will be used to deal with the non-compliance;
4) Analyses will be conducted primarily using the SPSS software, version 25.0 (IBM Corp., Chicago, IL, USA). Figures will be drawn using GraphPad Prism version 7.0.0 (GraphPad Software, San Diego, CA).

4.2 Data quality control

All responsible data collectors will be trained at the beginning of this study. An Electronic data capture (EDC) system will be used for data entry and management. The coordinator ensures that all data needed are collected.

4.3 Trial profile

The flow chart of inclusion and follow-up will be displayed in a diagram. The report will include the number of patients who met the inclusion criteria and the number included and reasons for exclusion of the non-included patients.

4.4 Patients characteristics

Description of the following baseline characteristics will be presented. Discrete variables will be summarized by frequencies and percentages. Percentages will be calculated according to the number of patients for whom the data are available. The number of missing values will be added in a footnote in the corresponding summary table. Continuous variables will be summarized by the use of standard measures of central tendency and dispersion, either mean and standard deviation [Mean ± SD], or median and 25%, 75% quartiles [Median(Q1-Q3)]. No statistical inference will be performed for the baseline variables. Baseline measures for all patients will be tabulated.

4.5 Patients drop out

The intention-to-treat principle will be used to deal with the patients who dropped out.

CONSORT 2010 checklist of information to include when reporting a randomised trial*

Section/Topic	Item No	Checklist item	Reported on page No
**Title and abstract**			
	1a	Identification as a randomised trial in the title	1
	1b	Structured summary of trial design, methods, results, and conclusions (for specific guidance see CONSORT for abstracts)	5
**Introduction**			
Background and objectives	2a	Scientific background and explanation of rationale	6-7
	2b	Specific objectives or hypotheses	7
**Methods**			
Trial design	3a	Description of trial design (such as parallel, factorial) including allocation ratio	17-18
	3b	Important changes to methods after trial commencement (such as eligibility criteria), with reasons	17-18
Participants	4a	Eligibility criteria for participants	17
	4b	Settings and locations where the data were collected	17
Interventions	5	The interventions for each group with sufficient details to allow replication, including how and when they were actually administered	18-19
Outcomes	6a	Completely defined pre-specified primary and secondary outcome measures, including how and when they were assessed	20-22
	6b	Any changes to trial outcomes after the trial commenced, with reasons	21
Sample size	7a	How sample size was determined	22
	7b	When applicable, explanation of any interim analyses and stopping guidelines	NA
Randomisation:			
Sequence generation	8a	Method used to generate the random allocation sequence	18
	8b	Type of randomisation; details of any restriction (such as blocking and block size)	18
Allocation concealment mechanism	9	Mechanism used to implement the random allocation sequence (such as sequentially numbered containers), describing any steps taken to conceal the sequence until interventions were assigned	18
Implementation	10	Who generated the random allocation sequence, who enrolled participants, and who assigned participants to interventions	18

Blinding	11a	If done, who was blinded after assignment to interventions (for example, participants, care providers, those assessing outcomes) and how	18
	11b	If relevant, description of the similarity of interventions	NA
Statistical methods	12a	Statistical methods used to compare groups for primary and secondary outcomes	22-23
	12b	Methods for additional analyses, such as subgroup analyses and adjusted analyses	22-23
**Results**			
Participant flow (a diagram is strongly recommended)	13a	For each group, the numbers of participants who were randomly assigned, received intended treatment, and were analysed for the primary outcome	36
	13b	For each group, losses and exclusions after randomisation, together with reasons	36
Recruitment	14a	Dates defining the periods of recruitment and follow-up	8
	14b	Why the trial ended or was stopped	8
Baseline data	15	A table showing baseline demographic and clinical characteristics for each group	32
Numbers analysed	16	For each group, number of participants (denominator) included in each analysis and whether the analysis was by original assigned groups	8
Outcomes and estimation	17a	For each primary and secondary outcome, results for each group, and the estimated effect size and its precision (such as 95% confidence interval)	8-11
	17b	For binary outcomes, presentation of both absolute and relative effect sizes is recommended	8-11
Ancillary analyses	18	Results of any other analyses performed, including subgroup analyses and adjusted analyses, distinguishing pre-specified from exploratory	NA
Harms	19	All important harms or unintended effects in each group (for specific guidance see CONSORT for harms)	10
**Discussion**			
Limitations	20	Trial limitations, addressing sources of potential bias, imprecision, and, if relevant, multiplicity of analyses	15
Generalisability	21	Generalisability (external validity, applicability) of the trial findings	13-15
Interpretation	22	Interpretation consistent with results, balancing benefits and harms, and considering other relevant evidence	13-15
**Other information**			
Registration	23	Registration number and name of trial registry	5
Protocol	24	Where the full trial protocol can be accessed, if available	23
Funding	25	Sources of funding and other support (such as supply of drugs), role of funders	30

*We strongly recommend reading this statement in conjunction with the CONSORT 2010 Explanation and Elaboration for important clarifications on all the items. If relevant, we also recommend reading CONSORT extensions for cluster randomised trials, non-inferiority and equivalence trials, non-pharmacological treatments, herbal interventions, and pragmatic trials. Additional extensions are forthcoming: for those and for up to date references relevant to this checklist, see www.consort-statement.org.
