# Supplementary material for: Ulinastatin in the treatment of radiotherapy-induced oral mucositis in locoregionally advanced nasopharyngeal carcinoma: a phase 3 randomized clinical trial
Source: Nat Commun. 2025 Mar 23;16:2848. doi: 10.1038/s41467-025-57884-6 (PMC11930952; doi:10.1038/s41467-025-57884-6)
Supplement: Supplementary file 2 — Reporting Summary [file 41467_2025_57884_MOESM2_ESM.pdf]

nature portfolio

Corresponding author(s): Lin Wang
Last updated by author(s): Feb 25, 2025

Reporting Summary

Nature Portfolio wishes to improve the reproducibility of the work that we publish. This form provides structure for consistency and transparency in reporting. For further information on Nature Portfolio policies, see our Editorial Policies and the Editorial Policy Checklist.

Statistics

For all statistical analyses, confirm that the following items are present in the figure legend, table legend, main text, or Methods section.

n/a	Confirmed	
☐	☒	The exact sample size (n) for each experimental group/condition, given as a discrete number and unit of measurement
☒	☐	A statement on whether measurements were taken from distinct samples or whether the same sample was measured repeatedly
☐	☒	The statistical test(s) used AND whether they are one- or two-sided<br>*Only common tests should be described solely by name; describe more complex techniques in the Methods section.*
☒	☐	A description of all covariates tested
☒	☐	A description of any assumptions or corrections, such as tests of normality and adjustment for multiple comparisons
☐	☒	A full description of the statistical parameters including central tendency (e.g. means) or other basic estimates (e.g. regression coefficient) AND variation (e.g. standard deviation) or associated estimates of uncertainty (e.g. confidence intervals)
☐	☒	For null hypothesis testing, the test statistic (e.g. F, t, r) with confidence intervals, effect sizes, degrees of freedom and P value noted<br>*Give P values as exact values whenever suitable.*
☒	☐	For Bayesian analysis, information on the choice of priors and Markov chain Monte Carlo settings
☒	☐	For hierarchical and complex designs, identification of the appropriate level for tests and full reporting of outcomes
☒	☐	Estimates of effect sizes (e.g. Cohen's d, Pearson's r), indicating how they were calculated

Our web collection on statistics for biologists contains articles on many of the points above.

Software and code

Policy information about availability of computer code

Data collection	The data in this study were collected using the Patient Electronic Medical Records System (Realone, Neusoft [version 2021.12]).
Data analysis	The data in this study were analyzed using SPSS (version 25.0).

For manuscripts utilizing custom algorithms or software that are central to the research but not yet described in published literature, software must be made available to editors and reviewers. We strongly encourage code deposition in a community repository (e.g. GitHub). See the Nature Portfolio guidelines for submitting code & software for further information.

Data

Policy information about availability of data

All manuscripts must include a data availability statement. This statement should provide the following information, where applicable:

- Accession codes, unique identifiers, or web links for publicly available datasets
- A description of any restrictions on data availability
- For clinical datasets or third party data, please ensure that the statement adheres to our policy

The data related to oral mucositis and oral pain generated in this study have been deposited in the DRYAD database under DOI: 10.5061/dryad.tb2rbp0ck.
The Clinical raw data are not publicly available due to involving patient privacy, but can be accessed on request from the corresponding author for 10 years. Any request should be sent to the corresponding author, L.W., via email at wangl1@sysucc.org.cn, along with a detailed description of your research protocol; individual deidentified participant data will be shared. Please allow one month for response to requests. The corresponding author and Sun Yat-sen University Cancer Center

will evaluate the reasonability of the request for our data and reserve the right to decide whether to share the data or not. And the data is only used for the research purpose. All data shared will be de-identified and will be available for 1 year after access is granted. The study protocol is available as Supplementary Note in the Supplementary Information file. The data related to oral mucositis and oral pain generated in this study are provided in the Source Data file. Source data are provided with this paper.

Research involving human participants, their data, or biological material

Policy information about studies with human participants or human data. See also policy information about sex, gender (identity/presentation), and sexual orientation and race, ethnicity and racism.

Reporting on sex and gender	Sex was self-report and was not considered in study design. The sex distribution was 127 (70.9%) men versus 52 (29.1%) women and this data was presented in the table of characteristics.
Reporting on race, ethnicity, or other socially relevant groupings	n/a
Population characteristics	Table 1 lists baseline characteristics of the population.
Recruitment	The participants were screened and enrolled into this trial by investigators from five participating treatment centers. Participants must meet the all the inclusion criteria and should not meet any one of the exclusion criteria as defined in the protocol. No other potential self-selection bias was present in this trial.
Ethics oversight	The study was approved by the Ethics Committee of Sun Yat-sen University Cancer Center (B2017-063-01-01) and registered on ClinicalTrials.gov, number NCT03387774. All patients provided written informed consent.

Note that full information on the approval of the study protocol must also be provided in the manuscript.

Field-specific reporting

Please select the one below that is the best fit for your research. If you are not sure, read the appropriate sections before making your selection.

☒ Life sciences ☐ Behavioural & social sciences ☐ Ecological, evolutionary & environmental sciences

For a reference copy of the document with all sections, see nature.com/documents/nr-reporting-summary-flat.pdf

Life sciences study design

All studies must disclose on these points even when the disclosure is negative.

Sample size	According to previous studies, the incidence of grade ≥ 3 oral mucositis in nasopharyngeal carcinoma patients treated by IMRT with cisplatin was about 40%. Assuming that the incidence of grade ≥ 3 RTOM in the Ulinastatin group decreased to 20%, the significance test level α= 0.05, Power= 0.8, at least 80 cases in each group were estimated. At least 176 patients (88 cases per group) were required to be enrolled in this study based on a 10% dropout and loss of follow-up rate.
Data exclusions	1) Prior anti-tumor therapy ; 2) metachronous or synchronous malignancy; 3) pregnant or lactating female; 4) pre-existing oral mucositis; 5) unwilling to discontinue harmful habits such as smoking, drinking alcohol and chewing betel nut; 6) severe comorbidities
Replication	This is a multicenter, open-label, randomized controlled clinical trial. Our finding can not be reproduced.
Randomization	Block randomization was performed at SYSUCC using a computer-generated random allocation sequence. Patients were randomly assigned before starting treatment to receive either Ulinastatin plus CCRT (UTI group) or CCRT alone (control group).
Blinding	After obtaining informed consent, the investigators at each institution contacted the study coordinators at the Clinical Trials Center and received treatment assignment information. The statistician (Y.G.) and study coordinators were uninvolved in the treatment of patients and data monitoring.

Reporting for specific materials, systems and methods

We require information from authors about some types of materials, experimental systems and methods used in many studies. Here, indicate whether each material, system or method listed is relevant to your study. If you are not sure if a list item applies to your research, read the appropriate section before selecting a response.

Materials & experimental systems

n/a	Involved in the study
☒	☐ Antibodies
☒	☐ Eukaryotic cell lines
☒	☐ Palaeontology and archaeology
☒	☐ Animals and other organisms
☐	☒ Clinical data
☒	☐ Dual use research of concern
☒	☐ Plants

Methods

n/a	Involved in the study
☒	☐ ChIP-seq
☒	☐ Flow cytometry
☒	☐ MRI-based neuroimaging

Clinical data

Policy information about clinical studies

All manuscripts should comply with the ICMJEguidelines for publication of clinical research and a completedCONSORT checklist must be included with all submissions.

Clinical trial registration: ClinicalTrials.gov Identifier: NCT03387774

Study protocol: Supplementary protocol in Supplementary information

Data collection: This trial was performed at five participating treatment centers in China. Patients were recruited between January 29, 2018, and December 28, 2021.

Outcomes: Primary endpoint of this study was the incidence of graded ≥ 3 acute RTOM in both groups. Secondary endpoints included the following: 1. cumulative incidence of grade ≥ 3 RTOM from 0 to 7 weeks of radiotherapy ; 2. onset time of grade ≥ 3 RTOM ; 3. duration of grade ≥ 3 RTOM from 0 to 7 weeks of radiotherapy ; 4. recovery rate (proportion of patients with grade ≥ 3 RTOM who recovered to grade ≤ 2 during CCRT); 5. numerical rating scale (NRS) pain scores; 6. safety; 7. completion rate of planned CCRT and the incidence of radiotherapy interruption (radiotherapy interruption ≥ 5 days); 8. overall survival (OS, durations were calculated from the date of randomization to the date of last follow-up or death from any cause), locoregional relapse-free survival (LRRFS, durations were calculated from the date of randomisation and the date of locoregional recurrence, or death from any cause), distant metastasis-free survival (DMFS, durations were calculated from the date of randomisation and the date of distant metastasis, or death from any cause) and progression-free survival (PFS, durations were calculated from the date of randomisation to the date of locoregional recurrence, distant metastasis, or death from any cause, whichever occurred first). Patients unavailable for follow-up or alive without distant metastasis or locoregional relapse were censored at the date of last follow-up.

The grade of RTOM and oral pain were recorded every week from the start of radiotherapy for each patient until the end of the seventh week. The acute toxicity criteria of the Radiation Therapy Oncology Group (RTOG) (scored by the physician) was used for RTOM assessment. Oral pain was assessed according to the NRS pain score. Acute chemotherapy toxic reactions during treatment were graded according to the Common Terminology Criteria for Adverse Events, version 5.0 (CTCAE v5.0). Radiotherapy toxicity was assessed according to the radiation damage grading criteria proposed by the RTOG.

Plants

Seed stocks: NA

Novel plant genotypes: NA

Authentication: NA
